# Synthesis of Marine Natural Products and Molecules Inspired by Marine Substances II

**DOI:** 10.3390/md19090518

**Published:** 2021-09-13

**Authors:** Emiliano Manzo

**Affiliations:** Institute of Biomolecular Chemistry (ICB), Consiglio Nazionale delle Ricerche (CNR), Via Campi Flegrei 34, 80078 Pozzuoli, Italy; emanzo@icb.cnr.it

The sea occupies more than 70% of the Earth’s surface and includes more than 300,000 organisms with huge biodiversity. These organisms represent an enormous source of substances with relevant potential in the pharmacological field. The diversity and potential bioactivity of structures isolated from marine organisms have always played a crucial role in the search for and discovery of novel bioactive molecules that are potentially useful in pharmacological applications. The development of new lead compounds from the sea has always been the crucial aim of drug discovery [1]. In this regard, the progress of chemical synthesis and synthetic strategies, leading to preparation and biological optimization of these substances, paves the way for new classes of biologically active compounds with pharmacological potential.

This Special Issue, as a continuation of the previous Special Issue, ‘Synthesis of Marine Natural Products and Molecules Inspired by Marine Substances’, includes three reviews and two articles that describe the synthetic strategies and biological activities of different classes of bioactive marine metabolites and analogs essential for future pharmacological applications.

A review by Munekata et al. [2] focuses on the development of synthetic chemical strategies for the preparation of marine alkaloid compounds and analogs to ameliorate the biological behavior of these substances. In particular, for the research and validation of the bioactivities, an increasing number of in vivo studies with animals, especially mice and zebrafish, have evidenced the potential health benefits against several important and debilitating patologies in vivo.

In a review by Kazmaier and Junk [3], cycloheptapeptides molecules as ilamycins, rufomycins, and cyclomarins, including unusual amino acids with important biological activities, are treated. These substances are specifically active against a range of mycobacteria with no significant activity towards other Gram-positive and Gram-negative bacteria or fungi. A wide range of these derivatives can be obtained by fermentation, while bioengineering also allows the synthesis of derivatives, especially cyclomarins. Other derivatives are accessible by semisynthesis or total syntheses, reported for both natural product classes. This review examines all aspects of the research conducted in the synthesis of these interesting cyclopeptides.

A review by Martinez et al. [4] focuses on divergent total synthesis strategies applied to the preparation of families of natural products using a common late-stage pluripotent intermediate and offering the opportunities for preparation of structurally related compounds. Aureol is the main topic of this review.

In an article of Aljahdali et al. [5], a docking study useful for the finding of new antiviral agents was performed. In this regard Walleye dermal sarcoma virus (WDSV), responsible for the induction of a multifocal benign cutaneous tumor, is particularly prevalent in some localities. Efficient therapeutics against this virus have not been developed yet. This study utilizes computer-aided drug design approaches to develop molecules through homology modeling, molecular docking, and ADMET methods, and three potential antiviral candidates, Friedlein, Phytosterols, and 1-Triacontanol are identified. 

In an article by Xiao et al. [6], the first total synthesis of marine natural product, (-)-majusculoic acid and its seven analogs, is performed. This strategy features the application of conformational and stereochemical controlled reactions. The promising anti-inflammatory activity of the synthetic derivatives is evaluated with significant effect. Moreover, they do not show cytotoxicity against RAW264.7 cells, indicating that they might be potential anti-inflammatory agents.

As guest editor, I am grateful to all the authors who contributed their excellent results to this Special Issue, all the reviewers who carefully evaluated the submitted manuscripts, and *Marine Drugs* for their support and kind help.
